# Comparative Validity and Reproducibility Study of Various Landmark-Oriented Reference Planes in 3-Dimensional Computed Tomographic Analysis for Patients Receiving Orthognathic Surgery

**DOI:** 10.1371/journal.pone.0117604

**Published:** 2015-02-10

**Authors:** Hsiu-Hsia Lin, Ya-Fang Chuang, Jing-Ling Weng, Lun-Jou Lo

**Affiliations:** 1 Assistant Research Fellow, Craniofacial Research Center, Chang Gung Memorial Hospital, Chang Gung University, Taoyuan, Taiwan; 2 Research Assistant, Craniofacial Research Center, Chang Gung Memorial Hospital, Chang Gung University, Taoyuan, Taiwan; 3 Professor, Department of Plastic and Reconstructive Surgery, and Craniofacial Research Center, Chang Gung Memorial Hospital, Chang Gung University, Taoyuan, Taiwan; Georgia Regents University, College of Dental Medicine, UNITED STATES

## Abstract

**Background:**

Three-dimensional computed tomographic imaging has become popular in clinical evaluation, treatment planning, surgical simulation, and outcome assessment for maxillofacial intervention. The purposes of this study were to investigate whether there is any correlation among landmark-based horizontal reference planes and to validate the reproducibility and reliability of landmark identification.

**Materials and Methods:**

Preoperative and postoperative cone-beam computed tomographic images of patients who had undergone orthognathic surgery were collected. Landmark-oriented reference planes including the Frankfort horizontal plane (FHP) and the lateral semicircular canal plane (LSP) were established. Four FHPs were defined by selecting 3 points from the orbitale, porion, or midpoint of paired points. The LSP passed through both the lateral semicircular canal points and nasion. The distances between the maxillary or mandibular teeth and the reference planes were measured, and the differences between the 2 sides were calculated and compared. The precision in locating the landmarks was evaluated by performing repeated tests, and the intraobserver reproducibility and interobserver reliability were assessed.

**Results:**

A total of 30 patients with facial deformity and malocclusion—10 patients with facial symmetry, 10 patients with facial asymmetry, and 10 patients with cleft lip and palate—were recruited. Comparing the differences among the 5 reference planes showed no statistically significant difference among all patient groups. Regarding intraobserver reproducibility, the mean differences in the 3 coordinates varied from 0 to 0.35 mm, with correlation coefficients between 0.96 and 1.0, showing high correlation between repeated tests. Regarding interobserver reliability, the mean differences among the 3 coordinates varied from 0 to 0.47 mm, with correlation coefficients between 0.88 and 1.0, exhibiting high correlation between the different examiners.

**Conclusions:**

The 5 horizontal reference planes were reliable and comparable for 3D craniomaxillofacial analysis. These reference planes were useful in standardizing the orientation of 3D skull models.

## Introduction

Traditional cephalometric analysis has long been used in orthodontic and orthognathic practice for diagnosis, treatment planning, and assessment of treatment results [1–4]. An accurate measurement system derived from specific reference points is crucial for this approach. The Frankfort horizontal plane (FHP) (porion-orbitale) and midsagittal reference plane based on the FHP are the most common planes used for determining skull orientation. Limitations arise from complicated overlapping of anatomic structures and asymmetric development, primarily because of the presentation of a complex 3-dimensional (3D) maxillofacial structure in 2-dimensional (2D) imaging [5, 6]. Three-dimensional cone-beam computed tomography (CBCT) has attracted considerable attention as a modern diagnostic tool because it can accurately visualize and analyze the 3D shape and position of soft and hard tissues. CBCT has been increasingly used in reconstructing virtual 3D craniofacial models, orthodontic treatment, and computer-aided orthognathic surgery for patients with maxillofacial deformity and malocclusion [7–9]. Three-dimensional image analysis can considerably expand the scope and improve the measurement accuracy, and a critical step for the aforementioned applications is identifying a valid reference plane system [10, 11]. Horizontal reference planes that can be defined using landmark points or the head position have been used [12, 13]. A horizontal plane defined by the natural head position has been used as a 3D cephalometric reference system for surgical planning and outcome assessment after orthognathic surgery [14, 15]. This method is cumbersome and requires an additional digital orientation device. It can be inconvenient to capture the neutral head position, and the plane is influenced by growth. By contrast, landmark-based reference planes are advantageous because they are not affected by the head position, the landmark points are constant and easily located in 3D images, and they are familiar to clinicians.

Various 3D FHPs have been defined and applied. Park et al, Kim et al and Gateno et al defined the FHP by using both sides of the porion and the left or right side of the orbitale to analyze craniofacial morphology [16–20]. Terajima et al and Song et al defined the FHP by using the right porion, left porion, and midpoint of the orbitale to measure 3D skeletodental orientation [8, 21]. Cheung et al and Damstra et al defined the FHP by using the right orbitale, left orbitale, and midpoint of the porion to develop a 3D cephalometric analysis system to assess dentofacial deformity [22, 23]. Wong et al defined the FHP by using the right orbitale, left orbitale, and left or right porion for 3D CBCT analysis [24]. The lateral semicircular canal plane (LSP) has been used for studying unicoronal synostosis and craniofacial deformities because it is considered to be least affected by the malformation [25, 26]. However, few studies have compared the distinct reference planes for 3D image analysis, and the results were varied. Based on a literature review, no study has reported an investigation of the intraobserver reproducibility and interobserver reliability in identifying the reference plane. Pelo et al compared the average FHP and LSP for 10 patients with facial asymmetry by using a 3D virtual craniofacial model obtained from CT imaging [27]. Vinchon et al presented the application of the LSP in patients with unicoronal synostosis [26]. Oh et al reported that the right or left FHP is an appropriate horizontal reference plane for evaluating an occlusal cant in 3D CT imaging [28].

This study investigated whether there is any systematic difference between landmark-based horizontal reference planes. The hypothesis of this study was that there is no difference between these reference planes in studying patients with facial symmetry or asymmetry. The aim was to test the hypothesis by performing statistical comparisons among the various landmark-oriented reference planes and complete the validation process to assess the reproducibility and reliability of landmark identification

## Materials and Methods

### Image acquisition

This was a retrospective study based on CBCT images of 30 randomly selected patients (12 male patients and 18 female patients), aged 18 to 38 years, who had undergone orthodontic treatment and orthognathic surgery in the Craniofacial Research Center of Chang Gung Memorial Hospital from 2012 to 2013. The inclusion criteria were healthy adult patients with skeletal class II or III malocclusion. Patients who had a history of facial trauma, hemifacial microsomia, craniosynostosis, degenerative or inflammatory conditions, or inadequate imaging data were excluded.

The 3D maxillofacial images were acquired preoperatively and postoperatively by using an i-CAT CBCT scanner (Imaging Sciences International, Hatfield, PA, USA), with the patients’ teeth in light contact condition. The specifications were as follows: extended field of view of 22 cm (height) × 16 cm (depth), 120 kV, 5 mA, 50 Hz, scanning time of 40 seconds, and voxel size of 0.4 × 0.4 × 0.4 mm. The obtained data were then exported in Digital Imaging and Communications in Medicine (DICOM) format. The exported data were reconstructed to produce 3D models by using SimPlant Pro Crystal (Materialize Dental, Leuven, Belgium) in preparation for the 3D craniomaxillofacial analysis. The image data were stored in the Imaging Laboratory of the Craniofacial Research Center. The study protocol was approved by the Institutional Review Board of the authors’ organization (IRB No. 100–2842B). All participants in this study provided written informed consent. The Institutional Review Board approved this consent procedure. The patient depicted in the figures provided informed consent for the publication of his photograph.

### Study design

**A. Participant groupings.** All participants were divided into 3 groups, with 10 patients in each group, according to physical examination results. Groups 1, 2, and 3 comprised patients with facial symmetry, patients with apparent facial asymmetry, and patients with cleft lip and palate (CLP), respectively. Patients with CLP were placed in a separate group because they exhibited individual differences in morphology and structure and often differed considerably from the normal, including facial asymmetry.

**Fig 1:**
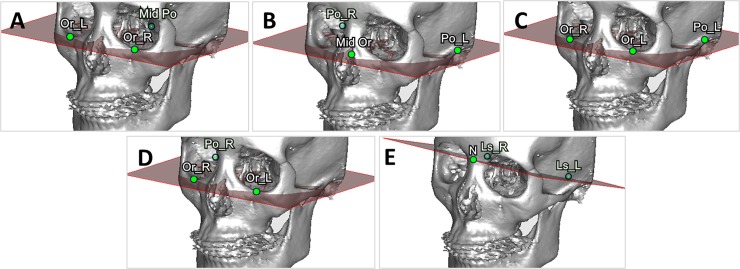
Various horizontal reference planes in the skull model. (A) FHP-P. (B) FHP-O. (C) FHP-L (D) FHP-R. (E) LSP.

**Fig 2:**
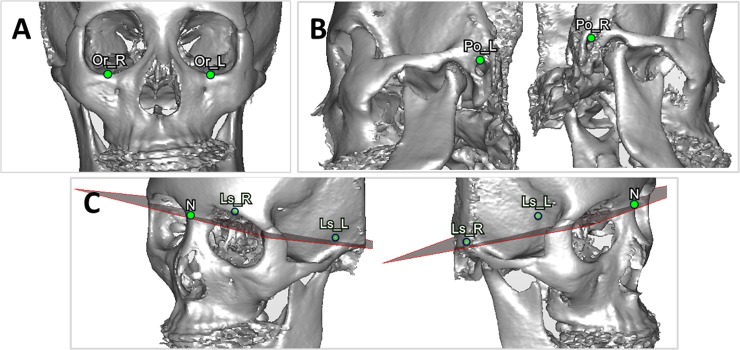
Landmark identification in the 3D model. (A) Identification of the orbitale point (red). (B) Identification of the left porion point (red). (C) Identification of the left lateral semicircular canal (red) in the axial plane.

**B. Definition of 3D landmarks and cephalometric-landmark-oriented reference planes.** Four non-coplanar points including the orbitales and porions were selected to define the FHP. Because 3 points define a plane, one option was to omit one of the points and another option was to average 2 paired points. A total of 5 planes were defined based on the identified landmark points and used for comparison (Fig. 1). The FHP-P plane passed through the average porion (PoA), right orbitale (OrR), and left orbitale (OrL). The FHP-O plane passed through the average orbitale (OrA), right porion (PoR), and left porion (PoL). The FHP-L plane passed through the PoL, OrR, and OrL; and the FHP-R plane passed through the PoR, OrR, and OrL. The LSP passed through the right lateral semicircular canal (LsR), left lateral semicircular canal (LsL), and nasion ponit (N). The orbitale point was defined as the most inferior point on the right (or left) infraorbital rim at which the tangent line was parallel to the horizontal line when the patient’s head position was adjusted (Fig. 2A). The porion point was defined as the highest point on the right (or left) external acoustic meatus; it was easily located when the patient’s head was rotated into a lateral view (Fig. 2B). The right (left) lateral semicircular canal was identified as an arc in the axial plane, and the most lateral point on the right lateral semicircular canal was selected as the LsR (LsL) point (Fig. 2C). These landmarks can be guided on the most appropriate CT slice in the axial, coronal, and sagittal view (S1 Fig.). Table 1 lists the definitions of the corresponding cephalometric landmarks in the 3D CBCT model.

**Table 1:** Definition of 3D cephalometric landmarks.

Landmark	Abbreviation	Definition
Right orbitale	OrR	The most inferior point of right infraorbital rim
Left orbitale	OrL	The most inferior point of left infraorbital rim
Right porion	PoR	The highest points of the right external acoustic meatus
Left porion	PoL	The highest points of the left external acoustic meatus
Average orbitale	OrA	Midpoint between the OrR and OrL.
Average porion	PoA	Midpoint between the PoR and PoL.
Right lateral semicircular canal	LsR	The most lateral point of the right lateral semicircular canal in the axial plane
Left lateral semicircular canal	LsL	The most lateral point of the left lateral semicircular canal in the axial plane
Nasion	N	The middle point of the frontonasal suture
Anterior nasal spine	ANS	The most anterior midpoint of the anterior nasal spine of the maxilla
Upper incisor	UI	Midoint between the crowns of the maxillary central incisors
Lower incisor	LI	Midpoint contact between the crowns of the mandibular central incisors.
Pogonion	Pog	The most anterior midpoint of the chin on the outline of the mandibular symphysis
Menton	Me	Menton is the most inferior midpoint of the chin on the outline of the mandibular symphysis.
Maxillary canine-left	UL3	Cusp of the left maxillary canine
Maxillary canine-right	UR3	Cusp of the right maxillary canine
Mandibular canine-left	LL3	Cusp tip of the left mandibular canine
Mandibular canine- right	LR3	Cusp tip of the right mandibular canine
First maxillary molar-left	UL6	Mesio-buccal cusp of the left first maxillary molar
First maxillary molar-right	UR6	Mesio-buccal cusp of the right first maxillary molar
First Mandibular molar-left	LL6	Mesio-buccal cusp of the left first maxillary molar
First Mandibular molar- right	LR6	Mesio-buccal cusp of the right first maxillary molar

**C. Validation of the intraobserver reproducibility and interobserver reliability of 3D landmark identification.** The precision of the landmark identification in all 3D CT models was evaluated by performing repeated tests, and the intraobserver reproducibility and interobserver reliability were assessed. The independent variables were point locations in the horizontal (X), vertical (Y), and transverse (Z) directions identified by intraobservers and interobservers.

**D. Comparison of various landmark-based reference planes.** The measurements of the Euclidean distance between the landmarks (Table 1) and 5 reference planes on each CBCT model were recorded to compare the differences among these planes. The independent variables were various horizontal planes based on 3D localization of facial landmarks in symmetric, asymmetric, and cleft conditions. The dependent variables were the differences between the right and left sides of different planes in terms of the distance of Euclidean space.

### Data collection methods

**A. Validation of the intraobserver reproducibility and intraobserver reliability of 3D landmark identification.** The accuracy of landmark identification was assumed to be high because the valid cephalometric reference plane system was determined using the landmarks. To validate the intraobserver reproducibility and interobserver reliability of landmark identification, 10 out of the 30 participants were randomly selected for examination. The landmarks comprising 14 previously defined points were located in the same participant by 2 observers twice after an interval of 1 week from the initial recording.

**Fig 3:**
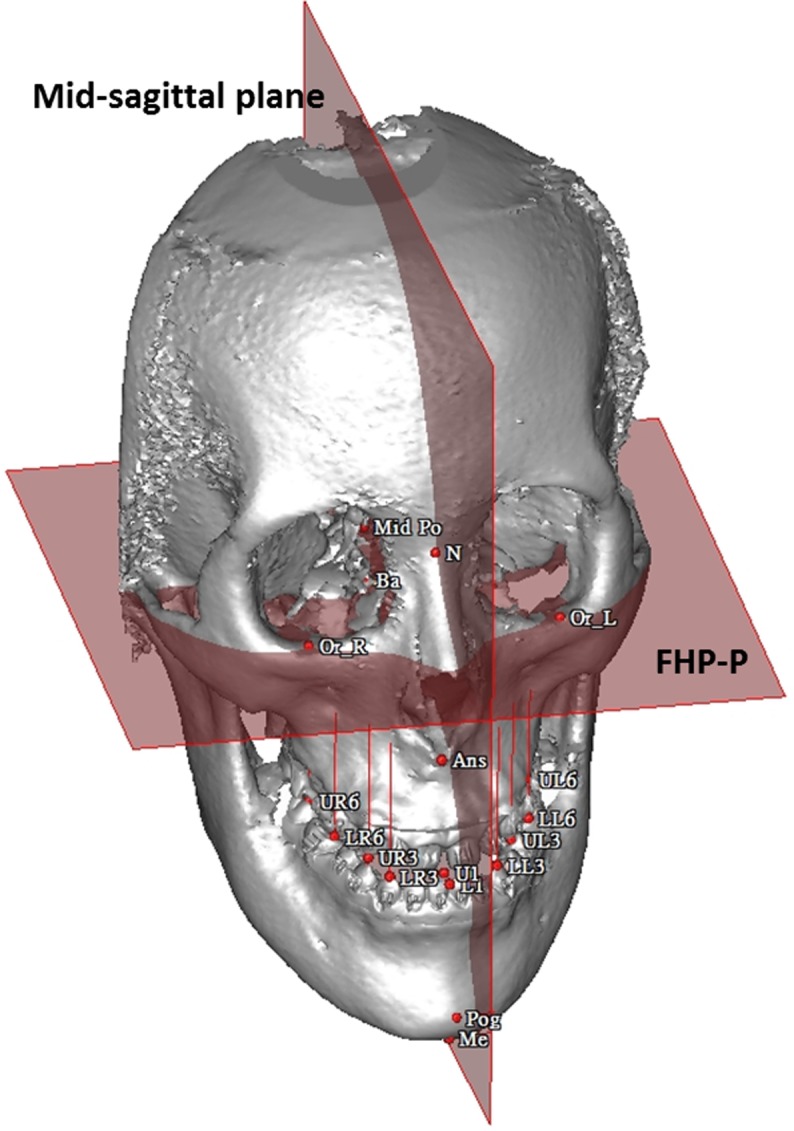
Example of measuring the distances from the reference plane, FHP-P, to the dental landmarks in a 3D CBCT model, and assessing postoperative symmetry according to the distance from the cephalometric points in the midline of the face to the mid-sagittal plane derived from the FHP-P.

**B. Comparison of various landmark-oriented reference planes.** Because the horizontal reference plane has long been used to evaluate the occlusal cant in 3D CT image, 8 points were selected from the maxillary and mandibular teeth (Table 1). The Euclidean distances between these points and each reference plane were measured (Fig. 3). The absolute differences between the 2 sides of distance were calculated and indicated as U3, L3, U6 and L6. The calculated differences were used to quantitatively compare the consistency among the 5 horizontal planes in each group.

### Data analysis

The obtained data were used for statistical analysis. Friedman’s test with a confidence level of 95% (p<.05) was used to compare the differences among the reference planes, and the Pearson correlation coefficient was determined to validate the intraobserver reproducibility and interobserver reliability. Statistical analyses were conducted using standard statistical software (SPSS Version 17, Chicago, IL, USA).

## Results

Group 1 comprised 3 men and 7 women (mean age 25.8 y); Group 2 comprised 4 men and 6 women (mean age 27.6 y); and Group 3 comprised 5 men and 5 women (mean age 20.3 y). Table 2 lists the patient information.

**Table 2:** Patient information.

Group	Subject	Age (years)	Sex	Diagnosis	Facial symmetry/ asymmtery
Group 1: patients with facial symmetry	1	21	F	Prognathism	Facial symmetry
	2	26	F	Prognathism with prominent angle and chin	Facial symmetry
	3	27	M	maxillary protrusion, mandible recession and gummy smile	Facial symmetry
	4	24	F	Prognathism	Facial symmetry
	5	31	F	Maxillary protrusion and anterior bite	Facial symmetry
	6	23	M	Prognathism	Facial symmetry
	7	22	M	Prognathism	Facial symmetry
	8	35	F	Prognathism	Facial symmetry
	9	27	F	Anterior open bite	Facial symmetry
	10	24	F	Prognathism	Facial symmetry
Group 2: patients with facial asymmetry	11	32	M	Prognathism and right chin deviation (6.6 mm)	Facial asymmetry
	12	30	M	Prognathism and right chin deviation (4.0 mm)	Facial asymmetry
	13	20	M	Prognathism and left chin deviation (5.7 mm)	Facial asymmetry
	14	30	F	Prognathism and left chin deviation (5.8 mm)	Facial asymmetry
	15	31	F	Prognathism and right chin deviation (4.1 mm)	Facial asymmety
	16	27	M	Prognathism and left chin deviation (4.2 mm)	Facial asymmetry
	17	31	F	Prognathism and left chin deviation (5.3 mm)	Facial asymmetry
	18	23	F	Prognathism and left chin deviation (7.0 mm)	Facial asymmetry
	19	21	F	Prognathism and right chin deviation (11.5 mm)	Facial asymmetry
	20	31	F	Prognathism and left chin deviation (8.8 mm)	Facial asymmetry
Group 3: patients with cleft lip/palate	21	20	M	Right cleft lip/palate	Facial asymmetry
	22	18	F	Bilateral cleft lip/palate	Facial asymmetry
	23	20	F	Left cleft lip/palate	Facial asymmetry
	24	21	F	Left cleft lip/palate	Facial asymmetry
	25	24	F	Cleft palate	Facial asymmetry
	26	21	M	Left cleft lip/palate	Facial asymmetry
	27	19	M	Left cleft lip/palate	Facial asymmetry
	28	22	M	Bilateral cleft lip/palate	Facial asymmetry
	29	19	M	Right cleft lip/palate	Facial asymmtery
	30	19	F	Right cleft lip/palate	Facial asymmetry

Physical examination demonstrated an asymmetry when chin deviation to one side was more than 4 mm

**A. Validation of the intraobserver reproducibility and interobserver reliability of 3D landmark identification.** The Pearson correlation coefficient showed no statistically significant difference in point location in the *X*, *Y* and *Z* directions within and between observers. For the intraobserver evaluation, the mean differences in the 3 directions varied from 0 to 0.35 mm, and the correlation coefficients (r) were between 0.96 and 1.0 (Table 3), exhibiting high correlation between these 2 data sets. For the interobserver test, the mean differences in the 3 directions varied from 0 to 0.47 mm, and the correlation coefficients (r) were between 0.88 and 1.0 (Table 3), exhibiting high correlation among various examiners. The results confirmed satisfactory intraobserver reproducibility and interobserver reliability in identifying the reference planes.

**Table 3:** Intraobserver reproducibility and interobserver reliability of 3D landmark identification.

	Landmark	X-axis			Y-axis			Z-axis		
		Mean	r	p-value	Mean	r	p-value	Mean	r	p-value
Intraobserver reproducibility	OrL	0.17	0.98	0.001*	0.14	0.99	0.003*	0.00	1.00	0.005*
	OrR	0.23	0.99	0.002*	0.00	0.99	0.008*	0.00	1.00	0.002*
	PoL	0.30	0.98	0.009*	0.07	0.99	0.002*	0.04	0.99	0.001*
	PoR	0.15	0.99	0.004*	0.02	0.99	0.003*	0.04	0.99	0.007*
	LsL	0.11	0.98	0.003*	0.14	0.97	0.001*	0.35	0.97	0.006*
	LsR	0.18	0.98	0.002*	0.27	0.96	0.004*	0.33	0.99	0.009*
	UL3	0.02	0.99	0.006*	0.06	0.99	0.002*	0.00	1.00	0.003*
	UR3	0.26	0.99	0.002*	0.35	0.98	0.001*	0.08	0.99	0.008*
	UL6	0.06	0.98	0.007*	0.03	0.96	0.001*	0.04	0.98	0.007*
	UR6	0.07	0.99	0.004*	0.05	0.99	0.005*	0.00	0.99	0.003*
	LL3	0.28	0.96	0.006*	0.13	0.99	0.003*	0.08	0.98	0.001*
	LR3	0.11	0.99	0.008*	0.02	0.97	0.009*	0.00	0.98	0.001*
	LL6	0.28	0.99	0.004*	0.06	0.99	0.002*	0.12	0.99	0.002*
	LR6	0.21	0.99	0.001*	0.12	0.99	0.007*	0.08	0.99	0.006*
Interobserver reliability	OrL	0.09	1.00	0.008*	0.06	0.91	0.002*	0.11	0.98	0.005*
	OrR	0.47	0.97	0.006*	0.23	0.88	0.004*	0.05	1.00	0.007*
	PoL	0.43	0.88	0.003*	0.47	0.93	0.006*	0.26	0.94	0.006*
	PoR	0.13	0.94	0.004*	0.16	0.96	0.003*	0.29	0.94	0.008*
	LsL	0.32	1.00	0.005*	0.33	0.90	0.006*	0.42	0.99	0.004*
	LsR	0.04	0.96	0.008*	0.31	0.89	0.006*	0.20	0.98	0.009*
	UL3	0.29	0.99	0.001*	0.04	0.90	0.006*	0.30	0.95	0.002*
	UR3	0.04	0.88	0.009*	0.27	0.90	0.003*	0.23	0.92	0.006*
	UL6	0.11	0.96	0.005*	0.02	0.98	0.003*	0.46	0.95	0.004*
	UR6	0.38	0.90	0.003*	0.31	0.91	0.006*	0.27	0.93	0.002*
	LL3	0.16	0.99	0.005*	0.16	0.99	0.001*	0.01	0.94	0.003*
	LR3	0.34	0.95	0.003*	0.19	0.96	0.008*	0.44	0.91	0.004*
	LL6	0.05	0.90	0.001*	0.45	1.00	0.002*	0.19	0.95	0.008*
	LR6	0.13	0.97	0.003*	0.46	0.91	0.008*	0.36	0.90	0.007*

r is Pearson correlation coefficient and

*correlation is highly significant at p≤.05.

**Fig 4:**
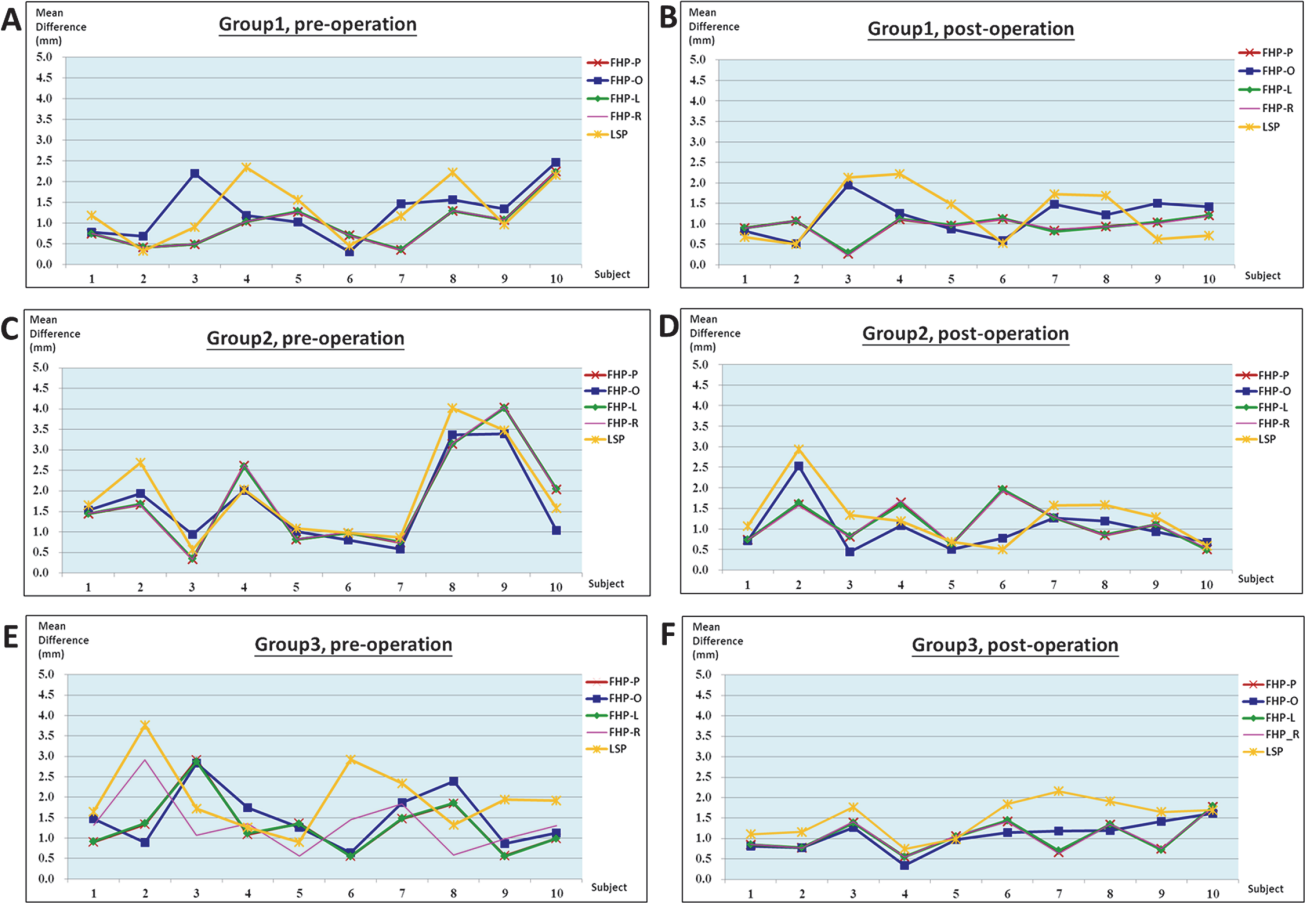
Discrepancies between the right and left distance from landmarks of maxillary and mandibular teeth to 5 reference planes for all participants. (A) Group 1: before surgery. (B) Group 1: after surgery. (C) Group 2: before surgery. (D) Group 2: after surgery. (E) Group 3: before surgery. (F) Group 3: after surgery.

**B. Comparison of various landmark-oriented reference planes.** The mean differences between the right and left distance from the maxillary and mandibular teeth landmarks to the 5 reference planes showed no statistically significant variation (p>.05) for all groups. In group 1, the mean difference ranged from 0.55 to 1.71 mm with a mean value of 0.93 mm. In goup 2, the mean difference ranged from 0.43 to 2.58 mm with a mean value of 1.45 mm. In group 3, the mean difference ranged from 0.64 to 2.25 mm with a mean value of 1.22 mm. Table 4 presents the results, and Fig. 4 illustrates the measurements of all participants for each preoperation and postoperation group.

**Table 4:** Mean differences between the right and left distance from landmarks of maxillary and mandibular teeth to the 5 reference planes.

Group	Plane	Pre-operation				Post-operation			
		U3	U6	L3	L6	U3	U6	L3	L6
		Mean±SD	Mean±SD	Mean±SD	Mean±SD	Mean±SD	Mean±SD	Mean±SD	Mean±SD
Group1	FHP-P	1.20±1.39	1.07±0.96	0.71±0.45	0.75±0.46	0.97±0.46	1.08±0.63	0.59±0.35	1.12±0.56
	FHP-O	1.39±1.32	1.4±0.99	0.74±0.65	0.94±0.48	1.02±0.48	1.49±0.93	0.71±0.53	1.43±0.77
	FHP-L	1.19±1.38	1.08±0.95	0.72±0.45	0.75±0.47	0.98±0.46	1.08±0.64	0.59±0.35	1.13±0.55
	FHP-R	1.02±1.40	1.07±0.97	0.72±0.44	0.75±0.46	0.97±0.47	1.09±0.64	0.59±0.35	1.12±0.58
	LSP	1.72±1.86	1.32±0.85	0.68±0.72	1.13±1.07	1.13±0.73	1.43±1.26	0.85±0.63	1.52±1.02
p-value		0.943	0.993	1.00	0.93	0.999	0.984	0.999	0.998
Group2	FHP-P	1.30±1.08	2.33±1.88	1.44±1.04	2.06±1.28	1.05±0.70	1.88±1.07	0.59±0.33	0.93±1.08
	FHP-O	1.16±0.98	2.27±1.71	1.29±0.78	1.93±1.24	0.82±0.63	1.71±1.04	0.43±0.28	1.08±1.36
	FHP-L	1.30±1.08	2.33±1.88	1.44±1.04	2.06±1.27	1.04±0.70	1.88±1.07	0.59±0.33	0.93±1.10
	FHP-R	1.31±1.08	2.32±1.89	1.45±1.04	2.06±1.29	1.05±0.71	1.89±1.06	0.58±0.34	0.93±1.06
	LSP	1.23±1.20	2.58±2.03	1.39±0.87	2.38±1.55	1.08±0.72	2.12±1.18	0.46±0.37	1.43±1.55
p-value		0.996	0.958	0.998	0.996	0.702	0.869	0.919	0.945
Group3	FHP-P	1.4±1.25	1.12±0.98	1.48±0.88	0.81±0.91	0.8±0.67	1.04±0.72	0.96±0.45	1.07±0.82
	FHP-O	1.56±1.13	1.61±0.91	1.5±0.95	1.23±1.09	0.87±0.64	0.99±0.62	0.98±0.56	1.02±0.83
	FHP-L	1.41±1.24	1.12±0.98	1.48±0.88	0.81±0.91	0.81±0.67	1.03±0.74	0.97±0.44	1.05±0.83
	FHP-R	1.41±1.24	1.12±0.98	1.48±0.88	0.81±0.90	0.80±0.67	1.05±0.71	0.96±0.45	1.08±0.81
	LSP	2.15±1.26	1.78±1.05	1.79±1.03	1.67±1.26	1.20±0.62	1.7±1.06	1.27±0.53	1.50±0.76
p-value		0.836	0.312	0.224	0.897	0.836	0.312	0.224	0.897

The differences between the right and left distance from landmarks of maxillary and mandibular teeth (8 landmarks defined in Table 2) to reference planes are indicated as U3:UL3−UR3; U6: UL6−UR6; L3: LL3−LR3; L6: LL6−LR6.

## Discussion

This study investigated whether there is an agreement among landmark-based horizontal reference planes. We hypothesized that there is no difference between these reference planes in patients with facial symmetry or asymmetry. The aim was to use statistical methods to compare the difference between the landmark-oriented reference planes and to validate the reproducibility and reliability of landmark indication by using 3D CBCT models. The results showed no statistically significant difference among the 5 reference planes in all groups, and the intraobserver reproducibility and interobserver reliability of landmark identification were excellent in this study; thus, the hypothesis was supported.

Although the 3D CBCT imaging system has been used for a long time, few studies have investigated whether the commonly used reference planes are reliable or comparable. Pelo et al conducted a study comparing the average FHP and LSP for 10 patients with facial asymmetry [27]; the results were similar to those obtained by Vinchon et al who reported that LSP is steady, reproducible, easily detectable, and more anatomical and functional [26]. Pelo et al showed that the discrepancy between FHP and LSP reference planes increases in cases with severe asymmetry, and decreases in patients with minor asymmetry. However, Pelo et al did not validate the intraobserver reproducibility and interobserver reliability in the construction of reference planes for 3D cephalometric analysis. Oh et al reported that the right and left FHPs are the most appropriate horizontal reference planes for evaluating an occlusal cant in 3D CT imaging; however, they did not compare the FHP with other commonly used reference planes [28]. The contributions of our study are in justifying the agreement among various horizontal reference planes and in validating the high reproducibility and reliability of landmark indication (Table 2). Furthermore, we applied the constructed reference planes to the 3D craniomaxillofacial analysis for the evaluation, planning, surgical simulation, and outcome assessment of orthognathic surgery. These applications are illustrated as follows.

This 3D reference plane can be effectively applied in orthognathic surgery simulation. One patient (Participant No. 13) was a 20-year–old man with prognathism, chin deviation (5.7 mm to the left), and class III dental malocclusion. S2A Fig. illustrates the patient’s preoperative photos and 3D CBCT model. The treatment goals were to correct the discrepancy between the maxilla and mandible, to correct dental malocclusion, and to improve facial appearance. The surgical plan comprised bilateral sagittal split osteotomy in the ramus for a mandibular setback of 8 mm on the right side and 2 mm on the left side, and genioplasty for a 3-mm chin advancement. Computer-assisted surgical planning and simulation were performed. The 3D model of the virtual simulation result, using each reference plane, was exported in stereolithography format for comparison between the models. Two selected models were registered and then superimposed to evaluate their differences. The magnitude, direction, and location of the disagreement between the models were displayed in a color-scale plot (S2B Fig.). In addition, the discrepancy was measured by performing a quantification analysis. The dental points described in Table 2 were used to calculate the difference of each point to the reference planes. The differences ranged from 0.36 to 2.23 mm (Table 5), exhibiting a clinically acceptable difference between the superimposed models. These reference planes can be applied in virtual surgical planning and simulation to achieve similar clinical results.

**Table 5:** Differences of vertical distance from each point to the reference planes (mm).

Reference planes	UL3	UR3	UL6	UR6	LL3	LR3	LL6	LR6
FHP_O vs. FHP_P	0.96	0.82	0.69	0.87	0.66	0.96	0.95	1.02
FHP_O vs. FHP_L	0.38	0.45	0.71	0.47	0.52	0.64	0.87	0.36
FHP_O vs. FHP_R	1.29	1.31	0.92	1.28	1.63	1.35	2.23	1.18
FHP_R vs. FHP_L	1.48	1.67	0.93	1.82	2.23	1.77	1.71	1.55
FHP_R vs. FHP_P	0.87	0.63	0.41	0.59	0.77	0.82	0.57	0.67
FHP_L vs. FHP_P	0.91	1.07	0.69	1.2	0.75	1.12	0.96	1.14

**Fig 5:**
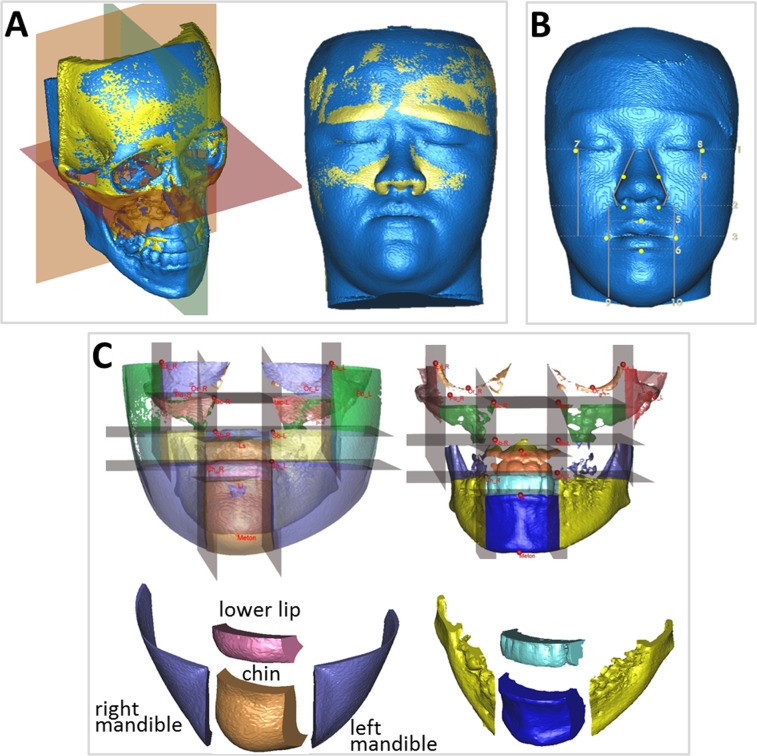
The volumetric difference of specific soft and hard tissue regions after surgery based on the constructed reference planes. (A) Cranial base superimposition of preoperative and postoperative CBCT models based on the reference planes (e.g., FHP-P). (B) The 3D landmarks were indicated, forming the horizontal and vertical borders of some specific regions based on the constructed reference planes. (C) The volumetric difference of the soft and hard tissue of various regions after subtracting the preoperative and postoperative images.

Another application is in surgical outcome assessment. One patient (Participant No. 19) was a 21-year-old woman with facial asymmetry with severe chin deviation of 11.5 mm to the right, a dental midline of 3 mm to the right, and greater midfacial height on the left. S2C Fig. shows the patient’s preoperative photos and 3D CBCT model. The treatment plan involved LeFort I maxillary osteotomy with 5-mm left posterior intrusion, 2-mm left anterior intrusion, 2-mm right posterior downward intrusion, bilateral sagittal split osteotomy of the ramus for a 0-mm setback on the right and 5-mm setback on the left, and genioplasty to implement a 5-mm shift to the left and a 2-mm lengthening. S2D Fig. illustrates the patient’s postoperative photos and 3D CBCT model. She was satisfied with the aesthetic results. For the outcome assessment, FHPs were used to evaluate postoperative symmetry according to the distance from the midline landmark points to the midsagittal plane derived from various horizontal reference planes, and to compare the differences among these planes (Fig. 3). The midsagittal plane was defined as the plane passing through the nasion and basion and perpendicular to the selected horizontal plane. The results showed significant improvement of facial symmetry and indicated no statistically significant difference among the reference planes (Table 6). Additional application was performed to compare the soft-to-hard tissue movement ratio in various facial regions and volumetric difference, using 5 reference planes in the 3D CBCT model after orthognathic surgery. First, the preoperative and postoperative CBCT models were superimposed based on the reference planes (Fig. 5A). Three-dimensional landmarks were then observed to form horizontal and vertical borders of specific regions, based on the constructed reference planes (Fig. 5B). The volumetric differences and the surfaces of each region were obtained and used to estimate the soft-to-hard tissue average movement ratio. Four regions were selected in this study—chin, right mandible, left mandible, and lower lip (Fig. 5C)—to compare the differences among the landmark-oriented reference planes. The mean value of the soft-to-hard tissue movement ratio in various facial regions for patients ranged from 0.74 to 1.07, which is a clinically acceptable ratio. The results indicated no statistically significant difference among the reference planes (p>.05) for all groups (Table 7).

**Table 6:** Comparison of reference planes on assessing facial symmetry.

	Distance of point to mid-sagittal plane (mm)		FHP-P	FHP-O	FHP-L	FHP-R
Pre-operation	ANS		+1.75	+1.12	+1.71	+1.80
	UI		+2.95	+1.99	+2.88	+3.17
	LI		+7.66	+6.71	+7.59	+7.73
	Pog		+9.87	+8.68	+9.79	+9.95
	Me		+11.82	+10.31	+11.46	+11.63
	p-value	0.9				
Post-operation	ANS		+0.64	+0.34	+0.62	+0.67
	UI		+1.38	+0.92	+1.35	+1.41
	LI		+1.54	+1.10	+1.51	+1.57
	Pog		+3.14	+2.54	+3.11	+3.19
	Me		+3.32	+2.71	+3.28	+3.37
	p-value	0.6				

The mid-sagittal plane was defined as the plane passing the nasion and basion and perpendicular to each FHP. Table 1 lists the definition of landmarks ANS, UI, LI, Pog, and Me. The positive sign indicates that the landmarks were located on the right side of the mid-sagittal plane. The p value was calculated from the distance groups to represent the difference among various planes.

**Table 7:** The soft-to-hard tissue movement ratio in different regions based on the different reference planes.

Group	Plane	Chin	Right mandible	Left mandible	Lower lip
		Mean±SD	Mean±SD	Mean±SD	Mean±SD
Group1	FHP-P	0.83±0.11	0.80±0.21	0.82±0.17	1.00±0.10
	FHP-O	0.76±0.49	0.75±0.38	0.79±0.28	1.02±0.33
	FHP-L	0.82±0.21	0.81±0.24	0.83±0.37	1.03±0.31
	FHP-R	0.81±0.13	0.79±0.33	0.85±0.39	1.01±0.22
	LSP	0.80±0.18	0.73±0.41	0.76±0.78	1.07±0.34
p-value		0.911	0.946	0.963	0.876
Group2	FHP-P	080.±0.39	0.76±0.33	0.83±0.11	1.01±0.22
	FHP-O	0.74±0.41	0.72±0.21	0.79±0.39	1.03±0.31
	FHP-L	0.81±0.24	0.80±0.18	0.80±0.15	1.01±0.45
	FHP-R	0.82±0.23	0.82±0.23	0.84±0.23	1.02±0.38
	LSP	0.79±0.36	0.80±0.19	0.78±0.89	1.05±0.37
p-value		0.782	0.828	0.720	0.769
Group3	FHP-P	0.79±0.18	0.80±0.41	0.83±0.33	1.03±0.19
	FHP-O	0.75±0.29	0.76±0.55	0.79±0.21	1.07±0.44
	FHP-L	0.80±0.17	0.83±0.22	0.81±0.22	1.02±0.28
	FHP-R	0.82±0.20	0.81±0.27	0.83±0.41	1.00±0.39
	LSP	0.81±0.31	0.79±0.38	0.80±0.25	1.02±0.25
p-value		0.211	0.223	0.458	0.571

This study had some limitations. Case 3 in Group 1 showed a higher difference between the right and left sides (Figs. 4A and 4B), and the discrepancy between the FHP-O plane and other FHPs was 1.7 mm. Repeated measurements of the vertical distance from the PoR and PoL points to other FHPs were performed, and the difference between these 2 points ranged from 1.31 to 1.33 mm. Case 7 in Group 3 was an outlier (Fig. 4F), and the discrepancy between the LS plane and other FHPs was greater than 1.5 mm. Measurements were repeatedly performed and confirmed. These phenomena might have been primarily caused by the large discrepancy between the external PoR and PoL landmarks, and by the internal LsR and LsL landmarks being in the vertical direction caused by the anatomical deviance of the patient. Generally, the 5 planes can be used with reliable consistency even in patients with facial asymmetry, as shown in Group 2. Outliers might have occurred. The 3D FHP-P, FHP-L, and FHP-R planes, which apply both anterior orbitale points, are recommended for facial bone planning and assessment to avoid possible outliers even though the statistical analysis in this study did not show significant differences among the planes. Prominent traumatic or congenital deformity of which related structures and landmark points are displaced, distorted, or absent causes a limitation in using these reference planes. Patients with hemifacial microsomia are examples, and in such cases, the horizontal reference plane must be redefined.

The strengths of this study include the demonstration that the proposed horizontal reference planes can be reliably used for preoperative and postoperative evaluation of craniofacial morphology on 3D CBCT models. The differences among these reference planes were comparable, as demonstrated by the patients shown in Fig. 4. Researchers can select a preferred or appropriate plane for their studies. The LS plane may be preferable in cranial and skull-base evaluations. The FHPs, particularly the 2 orbital points, are ideal for middle and lower face assessment as a continuation from the traditional 2D to the current 3D imaging system. In clinical practice, we recommend applying 3D FHPs rather than 3D craniomaxillofacial analysis for evaluation, planning, surgical simulation, and outcome assessment of orthognathic surgery. Some programs currently do not provide a function to conveniently locate the midpoint between paired orbitale or porion points; therefore, the FHP-O and FHP-P are not applied when using the software.

## Conclusion

The results of using reference planes for 3D craniomaxillofacial analysis were reliable, consistent, and comparable. These planes are satisfactory for determining the standard orientation of 3D skull models regardless of initial head position. Each of the planes can be selected for the evaluation, planning, simulation, and outcome assessment of orthognathic surgery. Although these preliminary results are encouraging, additional clinical cases in each group are required and will be collected to validate the present technique in future studies.

## Supporting Information

S1 Fig The landmarks in the most appropriate CT slice in the axial, coronal, and sagittal views. (A) Identification of the orbitale point (red). (B) Identification of the porion point (red). (C) Identification of the left lateral semicircular canal (red) in the axial plane.
(TIF)

S2 Fig (A) Preoperative 2D photos and 3D CBCT models. (B) Superimposition of 2 virtual surgical simulation models on a color map. The color maps show the location, direction, and magnitude of the differences between these models: green (0–0.4 mm), yellow (0.4–0.8 mm), orange (0.8–1.2 mm), red (1.2–1.6 mm), and purple (1.6–2.0 mm). (C) A patient’s preoperative 2D photos and 3D CBCT models. (D) A patient’s postoperative 2D photos and 3D CBCT models.
(TIF)
