# Supplementary figures and images for: Comparative Validity and Reproducibility Study of Various Landmark-Oriented Reference Planes in 3-Dimensional Computed Tomographic Analysis for Patients Receiving Orthognathic Surgery
Source: PLoS One. 2015 Feb 10;10(2):e0117604. doi: 10.1371/journal.pone.0117604 (PMC4323243; doi:10.1371/journal.pone.0117604)

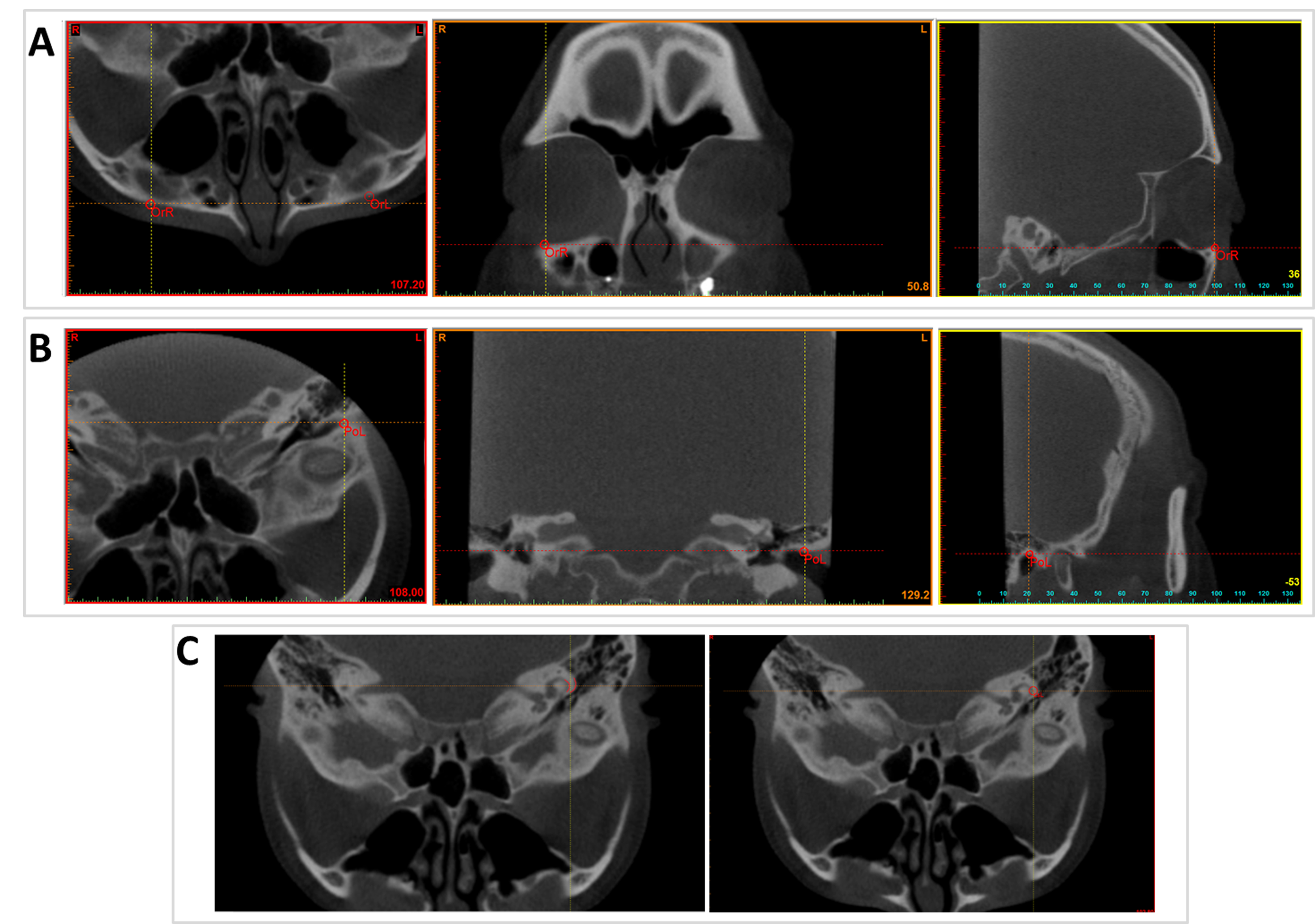

Supplement: S1 Fig — (A) Identification of the orbitale point (red). (B) Identification of the porion point (red). (C) Identification of the left lateral semicircular canal (red) in the axial plane. (TIF) [file pone.0117604.s001.tif]

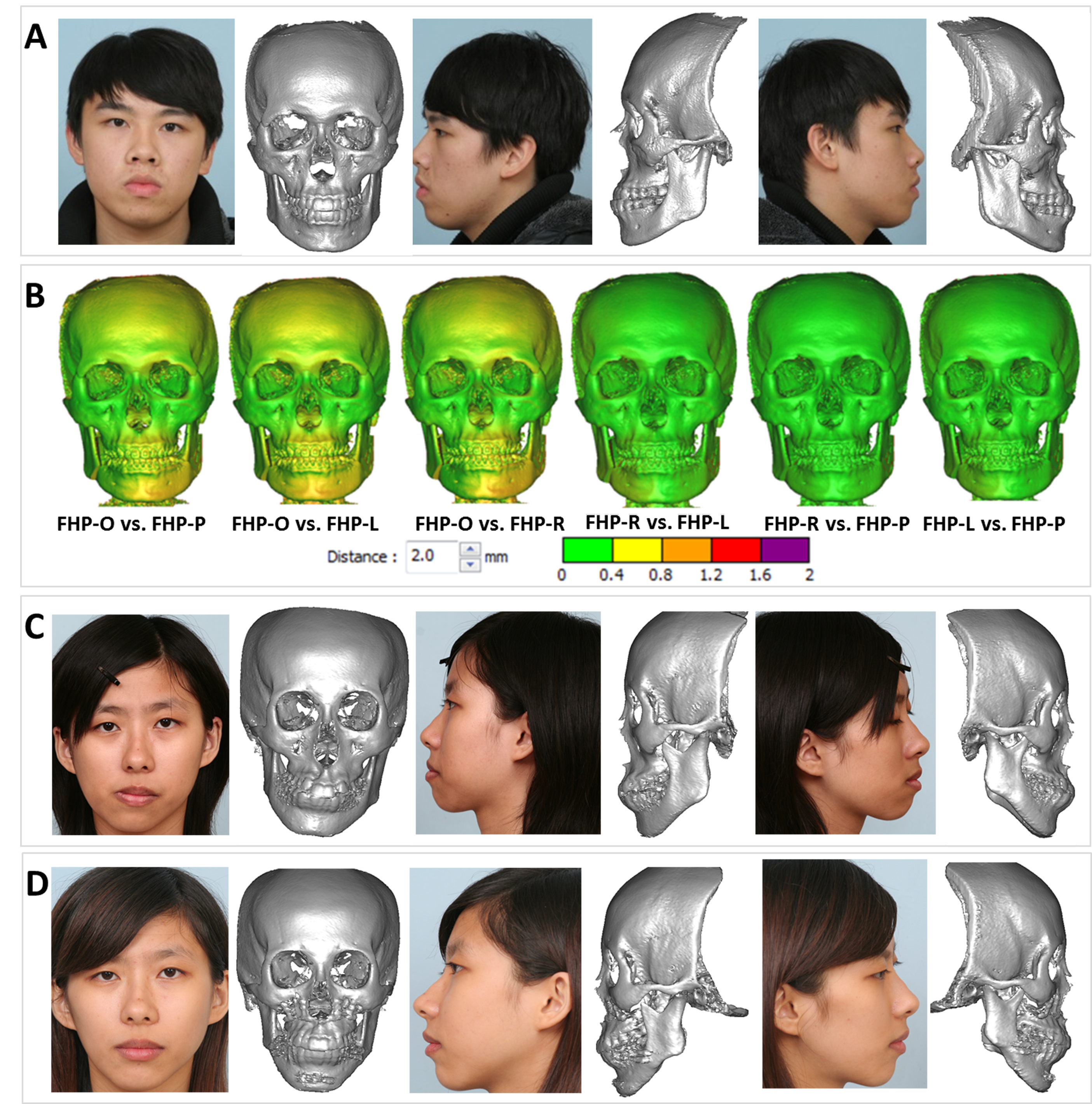

Supplement: S2 Fig — (B) Superimposition of 2 virtual surgical simulation models on a color map. The color maps show the location, direction, and magnitude of the differences between these models: green (0–0.4 mm), yellow (0.4–0.8 mm), orange (0.8–1.2 mm), red (1.2–1.6 mm), and purple (1.6–2.0 mm). (C) A patient’s preoperative 2D photos and 3D CBCT models. (D) A patient’s postoperative 2D photos and 3D CBCT models. (TIF) [file pone.0117604.s002.tif]
